# The global anaerobic metabolism regulator *fnr* is necessary for the degradation of food dyes and drugs by *Escherichia coli*

**DOI:** 10.1128/mbio.01573-23

**Published:** 2023-08-29

**Authors:** Lindsey M. Pieper, Peter Spanogiannopoulos, Regan F. Volk, Carson J. Miller, Aaron T. Wright, Peter J. Turnbaugh

**Affiliations:** 1 Department of Microbiology & Immunology, University of California, San Francisco, California, USA; 2 Biological Sciences Group, Pacific Northwest National Laboratory, Richland, Washington, USA; 3 Department of Biology, Baylor University, Waco, Texas, USA; 4 Department of Chemistry and Biochemistry, Baylor University, Waco, Texas, USA; 5 Chan Zuckerberg Biohub-San Francisco, San Francisco, California, USA; University of Michigan-Ann Arbor, Ann Arbor, Michigan, USA

**Keywords:** Human gut microbiome, xenobiotic metabolism, excipients, azoreductases, anaerobiosis, hydrogen sulfide, *Escherichia coli*, L-Cysteine, FNR, *fnrS*

## Abstract

The microbiome is an underappreciated contributor to intestinal drug metabolism with broad implications for drug efficacy and toxicity. While considerable progress has been made toward identifying the gut bacterial genes and enzymes involved, the role of environmental factors in shaping their activity remains poorly understood. Here, we focus on the gut bacterial reduction of azo bonds (R-N = N-R’), found in diverse chemicals in both food and drugs. Surprisingly, the canonical *azoR* gene in *Escherichia coli* was dispensable for azo bond reduction. Instead, azoreductase activity was controlled by the fumarate and nitrate reduction (*fnr*) regulator, consistent with a requirement for the anoxic conditions found within the gastrointestinal tract. Paired transcriptomic and proteomic analysis of the *fnr* regulon revealed that in addition to altering the expression of multiple reductases, FNR is necessary for the metabolism of L-Cysteine to hydrogen sulfide, enabling the degradation of azo bonds. Furthermore, we found that FNR indirectly regulates this process through the small noncoding regulatory RNA *fnrS*. Taken together, these results show how gut bacteria sense and respond to their intestinal environment to enable the metabolism of chemical groups found in both dietary and pharmaceutical compounds.

**IMPORTANCE:**

This work has broad relevance due to the ubiquity of dyes containing azo bonds in food and drugs. We report that azo dyes can be degraded by human gut bacteria through both enzymatic and nonenzymatic mechanisms, even from a single gut bacterial species. Furthermore, we revealed that environmental factors, oxygen, and L-Cysteine control the ability of *E. coli* to degrade azo dyes due to their impacts on bacterial transcription and metabolism. These results open up new opportunities to manipulate the azoreductase activity of the gut microbiome through the manipulation of host diet, suggest that azoreductase potential may be altered in patients suffering from gastrointestinal disease, and highlight the importance of studying bacterial enzymes for drug metabolism in their natural cellular and ecological context.

## INTRODUCTION

While it has long been appreciated that the gut microbiota, the trillions of microorganisms found in the gastrointestinal (GI) tract, and its aggregate genomes (the gut microbiome) contribute to the digestion and metabolism of dietary macronutrients, the broader role of the microbiome in the metabolism of xenobiotics (diet-derived and pharmaceutical small molecules) is less well understood. Recently, work in cell culture, mice, and humans has emphasized that both excipients (food and drug additives) and pharmaceuticals are extensively metabolized by the gut microbiota (1 – 6). The presence of an azo bond (R-N = N-R’) is shared between both pharmaceuticals and excipients (7, 8), which is notable given the ability of diverse human gut bacteria to reduce azo bonds (9). While azoreductase activity may be a core metabolic function of all human gut microbiotas (10 – 14), the enzymatic and nonenzymatic mechanisms responsible for this activity and their sensitivity to environmental factors remain poorly understood.

Mechanistic insights into this process are essential given the broad impact of azo reduction for antibiotics (15) and anti-inflammatory drugs (7, 16). Furthermore, the consumption of food, drug, and cosmetic (FD&C) dyes is increasing (17), providing additional substrates for gut bacterial metabolism. We recently discovered that FD&C dyes are potent inhibitors of the mammalian influx transporter OATP2B1, interfering with drug absorption in mice (18). This effect was rescued by human gut bacterial metabolism due to an inability of the downstream microbial metabolites to inhibit OATP2B1 (18). Additional work in rodent models has implicated FD&C dyes in carcinogenesis (19 – 21) and inflammatory bowel disease (22). Thus, the ability to predict or control azoreductase potential within the human gut microbiome could have broad implications for host health and disease.

The canonical enzyme implicated in this metabolic activity is azoreductase (AzoR) (9, 11, 14, 23). Work on the model human gut bacterium *Escherichia coli* has demonstrated that the purified AzoR protein is sufficient to reduce azo bonds (24). Other bacteria, for example, *Pseudomonas aeruginosa* (25 – 28) and *Enterococcus faecalis* (29 – 31) encode multiple azoreductases. Alternative mechanisms have been proposed for azoreductase activity, including the electron transport chain of *Shewanella oneidensis,* nicotinamide adenine dinucleotide (NADH), and hydrogen sulfide (H_2_S)(32 – 34). While these studies provide valuable mechanistic insight, a major limitation is their focus on *in vitro* biochemistry, neglecting to address the cellular and genetic mechanisms that impact azoreductase activity or the potential confounding effects of the complex physiological and microbiological interactions within the GI tract.

We sought to address this knowledge gap through the mechanistic dissection of a representative member of the human gut microbiota. Surprisingly, we found that the *azoR* gene is dispensable for the azoreductase activity of *E. coli*. In our search for alternative azoreductase enzymes, we discovered that *E. coli* azo reduction is regulated by the oxygen-sensing dual-transcriptional regulator, FNR. In turn, we show that FNR, via small regulatory RNA *fnrS*, regulates L-Cysteine metabolism, which produces H_2_S. Surprisingly, *E. coli*-produced H_2_S is sufficient to reduce azo dyes and drugs. Taken together, we demonstrate that environmental oxygen regulates the metabolism of L-Cysteine to H_2_S, which degrades commonly consumed azo-bonded compounds. Our results highlight the importance of studying and developing an understanding of the environmental context and regulation of key microbial metabolisms beyond annotated enzyme function.

## RESULTS

### *E. coli* AzoR is dispensable for azo dye depletion under anaerobic conditions

**Fig 1:**
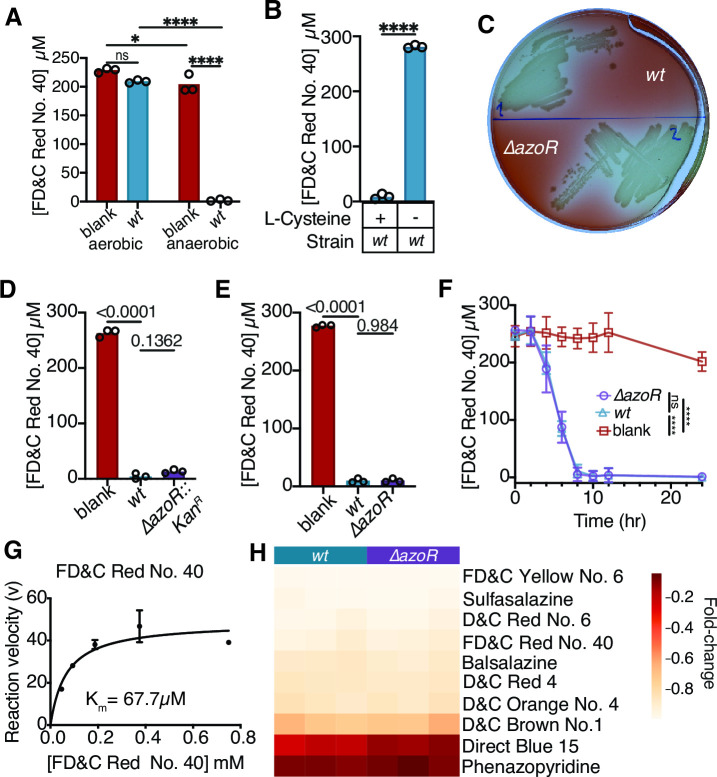
*E. coli* AzoR is sufficient but dispensable for azoreductase activity. (**A**) Azoreduction activity is significantly greater under anaerobic conditions. (**B**) Significant differences in FD&C Red No. 40 dye depletion is observed when *E. coli* is grown with or without L-Cysteine in the media. *E. coli azoR* is dispensable for azoreductase activity during anaerobic growth on (**C**) solid and (**D**) liquid media. (**E**) The knockout phenotype holds with a clean *azoR* deletion. (**A through **F and H) 250 µM FD&C Red No. 40. (**C**) 72 h growth on LB agar with 4.13 mM L-Cysteine. (**D and E**) 24 h growth in LB with 4.13 mM L-Cysteine. (**A and B, D and E**) Supernatant was removed from samples and analyzed for residual dye concentration spectrophotometrically. Concentrations calculated based on a standard curve, limit of detection = 1.2 µM. (**F**) Azoreduction activity over a 24 h time course. Media with 4.13 mM L-Cysteine and 250 µM FD&C Red No. 40 were inoculated with *wt* or *ΔazoR* and dye depletion was monitored over time. (**G**) Michaelis-Menten curve for purified AzoR enzyme with FD&C Red No. 40. Michaelis-Menten curve fit using PRISM. (**H**) Dye depletion in liquid media by *wt* or Δ*azoR* across a panel of azo drugs and dyes. Grown with 250 µM azo dye/drug, for 24 h in LB with 4.13 mM L-Cysteine. Fold changes are relative to average blank concentration. (**A and B, D **and E) Bars are mean. (F) Lines are mean ± stdev. (**A and B**) *P < 0.05, ****P < 0.0001, (**A and F**) two-way ANOVA, (**B**) Mann-Whitney test, (**D and E**) one-way ANOVA. (**A and B, D through H**) *n* = 3 biological replicates/strain.

Prior work on azoreduction in the context of the human gut and industrial settings has focused on anaerobic conditions (11, 23). We compared the depletion of a representative azo bond-containing food coloring (FD&C Red No. 40) by *E. coli* K-12 BW25113 under aerobic and anaerobic conditions, revealing that oxygen interferes with this activity (Fig. 1A). L-Cysteine, a commonly used supplement in anaerobic microbiology, was also required for FD&C Red No. 40 depletion by *E. coli* (Fig. 1B). Unless otherwise noted, these optimized culture conditions [Luria Broth (LB) medium plus 0.05% L-Cysteine] were used for our whole-cell azo dye depletion experiments.

Previously, we (18, 35) and others (36) had identified genes homologous to *E. coli* azoreductase (AzoR) in the genomes of diverse human gut bacteria capable of depleting drugs and other azo bond-containing compounds. We had originally sought to establish a tractable system to study these heterologously expressed genes by first abolishing this activity in *E. coli.* Much to our surprise, the *E. coli* Keio collection azoreductase knockout strain (Δ*azoR::kan^R^*) had activity indistinguishable from wild-type (*wt*) during growth in both solid (Fig. 1C) and liquid (Fig. 1D) media containing FD&C Red No. 40. We validated this finding by constructing and testing a clean deletion of *azoR* (Fig. 1E), which was confirmed by gel electrophoresis (Fig. S1A) and Sanger sequencing (Fig. S1B). The Δ*azoR* strain did not differ from *wt* in whole-cell dye depletion kinetics (Fig. 1F).

Given these unexpected results, we wondered if AzoR might be inactive in the tested *E. coli* strains, potentially due to mutations that could have occurred during the creation of our lab’s culture collection (37). Sanger sequencing of the *azoR* gene verified that the coding sequence was 100% identical to the deposited *E. coli* BW25113 genome. To further validate the activity of this enzyme, we purified the Histidine-tagged AzoR protein from *E. coli* AG1 with the pCA24N plasmid for overexpression (ASKA collection) (38). Cells were grown to mid-exponential growth phase, *azoR* expression was induced with isopropyl β-D-1-thiogalactopyranoside (IPTG), then cells were harvested and protein was purified using a nickel-charged affinity column. The fraction containing the most protein was determined using an SDS-PAGE gel and the size of the protein was consistent with AzoR (Fig. S2A). As expected, the purified protein was sufficient to clear a panel of azo bond-containing dyes and drugs (Fig. S2B). Additionally, we were able to create a Michaelis-Menten curve describing the enzymatic activity against FD&C Red No. 40, where we calculated a K_M_ of 67.7 µM (Fig. 1G). Finally, we tested our whole-cell assay using a panel of 10 food additives and pharmaceutical compounds containing azo bonds (Fig. 1H), demonstrating that *azoR* is unnecessary for the depletion of multiple dyes using the growth conditions established in Fig. 1A and B. These results confirm that the canonical AzoR enzyme in *E. coli* (24) is sufficient for azoreductase activity; however, *azoR* is not required for azo bond reduction by *E. coli* cells. These results emphasize that biochemical activity of purified proteins is not necessarily predictive of metabolism in whole cells and motivate a renewed search for the mechanism(s) responsible.

### FNR is necessary for the depletion of azo dyes by *E. coli*

**Fig 2:**
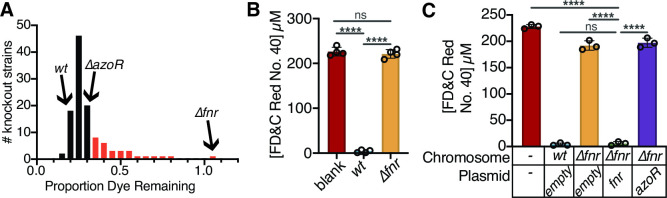
The *fnr* regulator is necessary for azoreductase activity in *E. coli*. (**A**) A targeted screen of 113 deletions of genes that encode enzymes and regulators revealed 22 loss-of-function strains (red bars, more dye remaining than the 95% confidence interval of the mean; *n* = 6 biological replicates/strain across two independent experiments). The Δ*fnr* (fumarate and nitrate reduction regulator) strain had the most extreme phenotype (complete loss-of-function). No significant gain-of-function phenotypes were observed. (**B**) Removal of the kanamycin cassette from Δ*fnr* still leads to a complete loss-of-function (*n* = 4 biological replicates/strain). (**C**) Complementation of *fnr* but not *azoR* rescues the azoreduction phenotype. Samples all induced with 0.1 mM IPTG at mid-exponential growth phase (*n* = 3 biological replicates/strain). All experiments: 24 h of growth in LB media, with 4.13 mM L-Cysteine and 250 µM FD&C Red No. 40, under anaerobic conditions. (**B and C**) Values are mean ± stdev. ****P < 0.0001, one-way ANOVA. Supernatant was removed from samples and analyzed for residual dye concentration spectrophotometrically. Concentrations calculated based on a standard curve. Limit of detection = 1.2 µM.

To identify genes necessary for azo dye depletion, we screened 113 candidate gene deletions from the Keio collection using a high-throughput liquid media assay for the depletion of the widely used food coloring FD&C Red No. 40. We focused on anaerobic reductases based on the requirement for anaerobic growth (Fig. 1A). We also included multiple genes based on prior studies linking them to azo dyes: genes upregulated by azo dyes in the presence of a quinone (39) or *S. oneidensis* MR-1 electron transport chain that is involved in azo dye depletion (40). Gene annotations were cross referenced on EcoCyc (41) (Table S1). Consistent with our previous data (Fig. 1), Δ*azoR* was comparable to *wt* (Fig. 2A; Table S1). A stringent cutoff of 95% confidence interval (CI) of all genes tested revealed 22 gene deletions that had significantly impaired activity (Fig. 2A). The most dramatic loss-of-function was found for Δ*fnr::Kan^R^* (fumarate and nitrate reduction regulator) resulting in a 2.3-fold decrease in azo dye depletion. We validated the role of *fnr* in FD&C Red No. 40 depletion by constructing a clean deletion (Fig. 2B) and confirming the deletion by gel electrophoresis (Fig. S1C) and Sanger sequencing (Fig. S1D). Activity was restored through complementation of *fnr* on the pCA24N plasmid from the ASKA collection (38) (Fig. 2C).

FNR plays a key role in oxygen sensing and the transition to anaerobic metabolism (42, 43), explaining the previously observed requirement for anaerobic conditions (Fig. 1A). The requirement for *fnr* was not unique to FD&C Red No. 40; significantly decreased dye depletion was observed across a panel of seven food dyes and three azo bond-containing drugs (Fig. S3). Overexpression of AzoR was insufficient to rescue Δ*fnr* (Fig. 2C), suggesting that other aspects of the vast *fnr* regulon (44 – 47) are responsible for the observed phenotype. Permeability issues likely result in inadequate dye reaching AzoR in the cytosol (48).

### Paired transcriptomics and proteomics reveals additional genes necessary for azo dye depletion

FNR has a large regulon that has been extensively characterized by transcriptomics (RNA-seq) and chromatin immunoprecipitation sequencing (ChIP-Seq) (43, 44, 46, 49). However, only 14 of the 55 genes found within the FNR regulon included in our genetic screen led to a significant loss-of-function at our 95% CI (Fig. 2A; Table S1), indicating that only a subset of the FNR regulon is necessary for azo dye depletion. Prior to testing additional candidate genes, we sought to confirm and extend prior work on the FNR regulon. Our culture conditions differ from past experiments in three ways that could alter gene expression and/or metabolic activity: (i) the presence of L-Cysteine; (ii) the inclusion of the azo dye FD&C Red No. 40; and (iii) the use of the vehicle DMSO, which is metabolized by *E. coli* under anaerobic conditions (50, 51). We also wanted to assess gene expression during stationary phase, given that FD&C Red No. 40 levels do not decrease until the exponential phase of growth (Fig. S4). Finally, we sought to build upon prior work on the FNR regulon by also assessing protein levels using proteomics.

**Fig 3:**
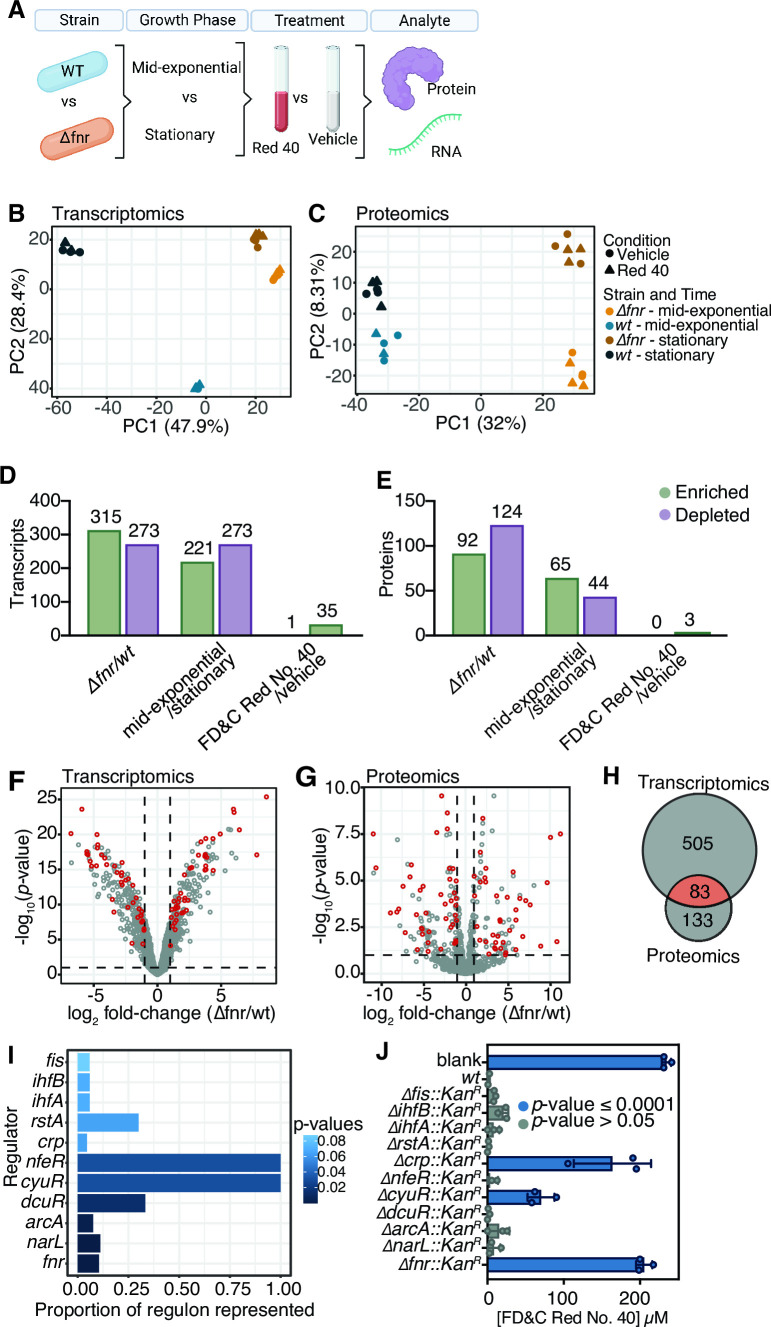
Multi-omic analysis of the *fnr* regulon reveals a complex regulon and the importance of L-Cysteine metabolism. (**A**) Experimental design. *wt* and *Δfnr* strains were grown to mid-exponential or stationary phase, then dosed with 250 µM FD&C Red No. 40 or vehicle control. After 45 min, cultures were spun down and fractions taken for proteomics or transcriptomics processing. Created with BioRender.com. (**B and C**) Principal components analysis (PCA) of Euclidean distances for transcriptomics (**B**) and proteomics (**C**) datasets. Each point represents one sample from the experiment. (**D and E**) Bar plots representing the number of features enriched or depleted (*p_adj_* <0.1, log_2_ fold change>|1|) for each comparison for transcriptomics (**D**) and proteomics (**E**) datasets. (**F and G**) Volcano plots for transcriptomics (**F**) and proteomics (**G**) datasets. Each point represents the average expression of one transcript or protein, respectively. Differential expression is based on the strain-level comparison. Significantly differentially expressed features (*p_adj_* <0.1, log_2_ fold change≥|1|) are indicated by dashed lines, comparing *Δfnr* to *wt*. (**H**) Venn diagram of the number of differentially expressed features (*p_adj_* <0.1, log_2_ fold change >|1|) in the two datasets. (**I**) Regulons enriched in the overlapping features of transcriptomics and proteomics datasets (*p_adj_* <0.1, with Benjamini-Hochberg correction) of differentially expressed genes (*p_adj_* <0.1, log_2_ fold change>|1|). (**J**) Azo depletion activity by enriched regulator knockout strains from the Keio Collection. Values are mean ± stdev.

We grew *wt* and Δ*fnr E. coli* in LB media with 0.05% L-Cysteine and inoculated the cultures with 250 µM FD&C Red No. 40 or DMSO vehicle at either mid-exponential or stationary growth phases (Fig. 3A). After a 40 min incubation period, samples were collected and split for paired transcriptomics (RNA-seq) and proteomics analysis. In total, we generated 12.4 ± 6.2 million high-quality reads/sample (RNA-seq; Table S2) and 881 ± 23 unique peptides after matching spectra through the MS-GF+ database (52) and adjusting for false discovery rate (FDR) of 0.01 against the MS-GF+ generated decoy database (proteomics; Table S3).

Principal coordinates analysis revealed a clear separation of profiles between strains at the RNA (Fig. 3B) and protein (Fig. 3C) levels. As expected (53 – 55), we also saw a clear difference in expression profile between growth phases; however, the separation between strains was maintained at both timepoints (Fig. 3B and C). There was a more minimal impact of FD&C Red No. 40 (Fig. 3B and C).

The number of differentially expressed genes was consistent with the overall trends in these datasets, revealing marked strain- and growth-phase differences in transcript and protein levels, with a minimal impact of FD&C Red No. 40 (Fig. 3D and E; Table S4). Only a single gene was significantly upregulated by FD&C Red No. 40 (log_2_ fold change >1 and *p*_adj_ <0.1): *ydeN*, a putative sulfatase (56, 57). The Δ*ydeN::KanR* strain had comparable azoreductase activity to *wt* (Fig. S5). In the proteomics data set three features were enriched in the vehicle group (Fig. 3E).

Given the marked difference in azo dye depletion between the *wt* and Δ*fnr* strains, we next focused on the 588 transcripts and 216 proteins significantly different between strains with a log_2_ fold change>|1| (*p_adj_* <0.1; Fig. 3F through H). Eighty-three features were both significant and had a fold change in the same direction in both datasets (Fig. 3H). Notably, these consistent features included 62 enzymes and 18 [oxido]reductases (Table S5). *azoR* was slightly significantly upregulated in the *wt* strain (Fig. S6A), with a trend toward increased peptide intensity (Fig. S6B). As expected, *fnr* transcripts were undetectable in the Δ*fnr* strain (Fig. S6C). Furthermore, 96/113 genes used in our azo dye depletion screen (Fig. 2A) had detectable expression (Table S1).

Given the size of the FNR regulon in both prior studies (43, 44, 46, 49) and our datasets, including numerous enzymes that could feasibly impact azo dyes, we sought to further narrow down our candidate gene list by focusing on FNR-dependent regulators. Features that were significantly increased in both RNA-seq and proteomics in *wt* compared to Δ*fnr* (log_2_ fold change>|1|, *p_adj_* <0.1) were analyzed for direct regulators using EcoCyc (see Materials and Methods). This analysis revealed 11 regulators whose regulons were significantly enriched in our gene set (Fig. 3I). As expected, *fnr* was the most significantly enriched regulator; however, the proportion of the FNR regulon represented was lower than some of the other regulators due to the much larger size of the FNR regulon and our stringent criteria for identifying FNR-dependent genes.

We went on to test the azoreduction phenotype of Keio collection knockouts of all 11 regulators (Fig. 3J). Two regulators that were not in our original screen, *cyuR* (detoxification of L-Cysteine regulator) and *crp* (cAMP-activated global transcriptional regulator), demonstrated a significant loss-of-function compared to *wt* (*P* < 0.0001, one-way ANOVA). Crp regulates >100 genes during both aerobic and anaerobic growth (58 – 61). In contrast, *cyuR* regulates just two genes involved in L-Cysteine metabolism: *cyuA* (L-Cysteine desulfidase) and *cyuP* (L-Cysteine utilization permease) (62 – 64). Given the tractable size of the *cyuR* regulon and emerging evidence that L-Cysteine metabolism can enable degradation of azo bonds (33, 34, 65), we opted to focus on *cyuA* and *cyuP*.

### L-Cysteine metabolism is required for depletion of azo dyes

**Fig 4:**
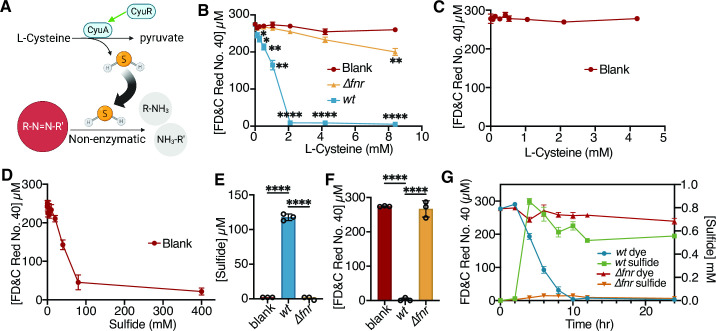
The *fnr* regulator is necessary for the metabolism of L-Cysteine to hydrogen sulfide, enabling the reduction of azo bonds. (**A**) Schematic depicting L-Cysteine metabolism pathway in *E. coli,* which metabolizes L-Cysteine to pyruvate, releasing hydrogen sulfide. Created with BioRender.com. (**B**) *wt* and *Δfnr E. coli* were incubated in various concentrations of L-Cysteine. Statistics relative to blanks. (**C**) Increasing L-Cysteine concentrations in LB with 250 µM FD&C Red No. 40. (**D**) Increasing sodium sulfide concentrations in LB with 250 µM FD&C Red No. 40 with 0.05% L-Cysteine. (**E**) Hydrogen sulfide production by *wt* and *Δfnr E. coli*. (**F**) FD&C Red No. 40 depletion by *wt* and *Δfnr E. coli*. (**G**) FD&C Red No. 40 depletion and sulfide measurements over time in *wt* (blue/green) and *Δfnr* (red/orange) *E. coli*. (**B, E and F**) *P < 0.05, **P < 0.01, ***P < 0.001, ****P < 0.0001, one-way ANOVA. All experiments: values are mean ± stdev. 24 h of growth in LB media, with 4.13 mM L-Cysteine and 250 µM FD&C Red No. 40 unless otherwise noted. Supernatant was removed from samples and analyzed for residual dye concentration spectrophotometrically. Concentrations calculated based on a standard curve. Limit of detection = 1.2 µM (*n* = 3 biological replicates).

L-Cysteine metabolism by *E. coli* generates hydrogen sulfide (H_2_S) as a dead-end metabolite (Fig. 4A) (63, 66), which has been previously implicated in azo dye degradation (33, 34, 65). Thus, we hypothesized that *fnr* controls H_2_S production and thus azo bond degradation by increasing the expression of the *cyuR*-regulated *cyuA* and *cyuP*. First, we tested the requirements for L-Cysteine and H_2_S in azoreduction. To facilitate rapidly establishing anaerobic conditions, we routinely supplement our media with L-Cysteine (67 – 69). Removal of L-Cysteine impaired the ability of *E. coli* to clear FD&C Red No. 40 (Fig. 1B). L-Cysteine promoted FD&C Red No. 40 depletion in a dose-dependent manner (Fig. 4B), suggesting that L-Cysteine is a key substrate for azoreductase activity during *in vitro* growth under anaerobic conditions. Anaerobic conditions in LB media lacking L-Cysteine, equilibrated in the anaerobic chamber for 1 wk were confirmed by eye using the indicator resazurin. Notably, L-Cysteine alone was unable to deplete FD&C Red No. 40 (Fig. 4C), while addition of sodium sulfide demonstrated a dose-dependent reduction in sterile media (Fig. 4D). To test the relationship between L-Cysteine and H_2_S, we quantified H_2_S in whole-cell culture using the methylene blue assay (see *Materials and Methods*). H_2_S levels were below our limit of detection (1.3 µM) in the Δ*fnr* strain, significantly less than the triple-digit µM levels produced by *wt* (Fig. 4E). We also quantified the amount of FD&C Red No. 40 depleted in these samples and saw the inverse trend (Fig. 4F). Consistent with the importance of L-Cysteine metabolism for dye depletion, peak H_2_S concentration corresponded to the decrease in FD&C Red No. 40 for *wt* with Δ*fnr* stable over time (Fig. 4G).

**Fig 5:**
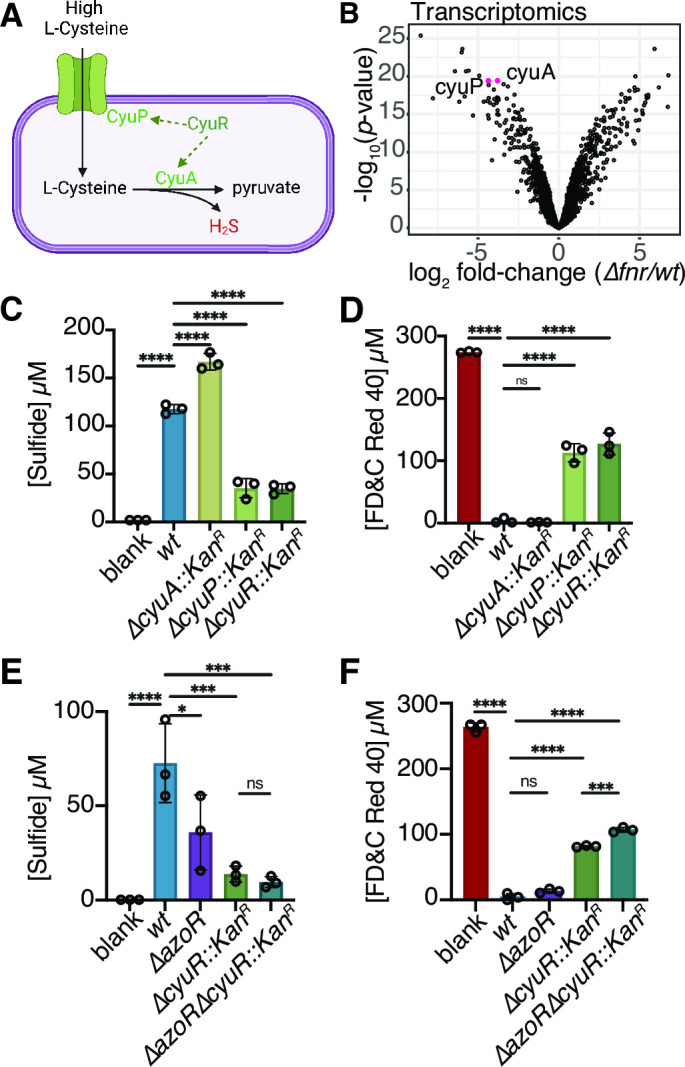
L-Cysteine import and metabolism genes impact bacterially produced sulfide and FD&C Red No. 40 depletion. (**A**) Model of the *cyuR* regulon’s role in L-Cysteine metabolism. CyuP acts as a L-Cysteine transporter into the cell, while CyuA is a L-Cysteine desulfidase. Created with BioRender.com. (**B**) Volcano plot for transcriptomics data: *cyuA* and *cyuP* are highlighted in pink. Each point represents the average expression of one transcript. Differential expression is based on the strain-level comparison. (**C and D**) Paired sulfide (**C**) and FD&C Red No. 40 (**D**) were measured from *cyuR* regulon knockouts and *cyuR* (Keio collection). (**E and F**) Paired sulfide (**E**) and FD&C Red No. 40 (**F**) were measured from *ΔazoR*, *ΔcyuR::Kan^R^*, and *ΔazoRΔcyuR::Kan^R^* strains. (C through F) Values are mean ± stdev. *P < 0.05, **P < 0.01, ***P < 0.001, ****P < 0.0001, one-way ANOVA (*n* = 3 replicates/strain).

Next, we investigated if H_2_S alone was the *fnr*-driven azoreductase mechanism for FD&C Red No. 40 depletion. We utilized single and multi-gene knockouts for the *cyuR* regulon. As previously mentioned, CyuR regulates both the L-Cysteine transporter (*cyuP*) and L-Cysteine desulfidase (*cyuA*) (63) (Fig. 5A). Consistent with our hypothesis, *cyuA*, *cyuP*, and *cyuR* were significantly decreased at the transcript level in *Δfnr E. coli* relative to *wt* controls (Fig. 5B; Fig. S6D). We validated these findings using RT-qPCR (Fig. S6E through G). The *cyuP* and *cyuR* knockout strains produced significantly less sulfide (Fig. 5C) and depleted significantly less FD&C Red No. 40 (Fig. 5D) in L-Cysteine-containing media. The combined deletion of *cyuR* and *azoR* did not significantly impact sulfide levels (Fig. 5E); however, there was a slight but statistically significant impairment in azo dye depletion (Fig. 5F). The addition of 0.05% L-Cysteine at mid-exponential phase increased the expression of *cyuR*, *cyuA*, and *cyuP; cyuR* and *cyuP* were induced significantly higher in *wt* relative to Δ*fnr* (Fig. S7A through D).

### FNR indirectly regulates the *cyu* genes

**Fig 6:**
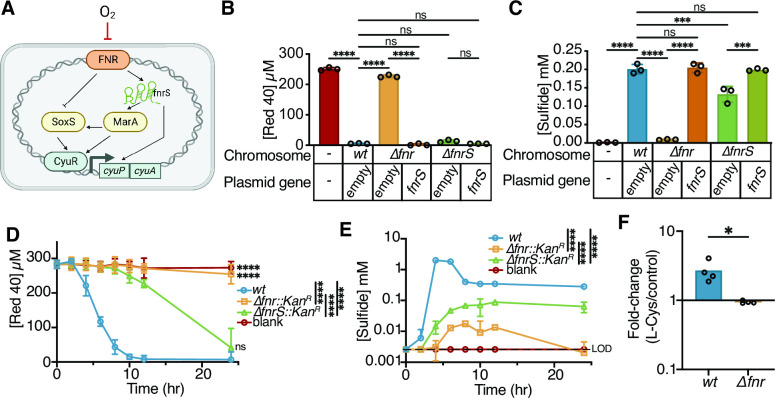
FNR indirectly regulates sulfide production and FD&C Red No. 40 depletion predominantly via small RNA *fnrS*. (**A**) Model of the potential regulation cascade from FNR to the expression of *cyuAP*. Created with BioRender.com. (**B and C**) Paired FD&C Red No. 40 (**B**) and sulfide (**C**) were measured from *wt*, *Δfnr::Kan^R^* complemented with pBD-lac or pBD-lac-fnrS, and *ΔfnrS::Kan^R^* complemented with pBD-lac or pBD-lac-fnrS. (**D and E**) Paired FD&C Red No. 40 (**D**) and sulfide (**E**) were measured from *wt*, *Δfnr::Kan^R^*, and *ΔfnrS::Kan^R^* over time. Samples inoculated at time point zero. (**E**) Plotted on a log_10_ scale y-axis. (**F**) Fold change of *fnrS* in the presence of L-Cysteine compared to vehicle at mid-exponential growth. All values are mean ± stdev. *P < 0.05, **P < 0.01, ***P < 0.001, ****P < 0.0001. (**B and C**) One-way ANOVA (*n* = 4 replicates/strain). (**D and E**) Two-way ANOVA for overall comparisons and two-way ANOVA comparing *wt* to each group at the final time point (*n* = 3 replicates/strain). (**F**) Mann-Whitney test (*n* = 4 biological replicates averaged from three technical replicates/strain).

Finally, we sought to better understand the mechanism through which FNR impacts the expression of *cyuR* and its target genes *cyuAP.* FNR was not detected at the *cyuR* promoter in chromatin immunoprecipitation experiments (44), consistent with the lack of the FNR binding motif TTGATnnnnATCAA (44). These results led us to hypothesize that FNR indirectly regulates the *cyu* genes. A review of the prior literature and the EcoCyc database identified multiple potential intermediates that are both under the control of FNR and affect *cyu:* the MarA and SoxS transcription factors (70) and the regulatory small noncoding RNA *fnrS* (71, 72) (Fig. 6A). MarA and SoxS both regulate *cyuR* (70), while *fnrS* is a proposed positive regulator of *cyuP* (72). Neither *marA* nor *soxS* complemented the Δ*fnr* phenotype (Fig. S7E and F). Furthermore, the Δ*marA::Kan^R^* and Δ*soxS::Kan^R^* strains were not significantly different from *wt* (Fig. S7G). Together, these experiments suggest that *marA* and *soxS* are not involved in the *fnr-*dependent FD&C Red No. 40 depletion or H_2_S production.

In contrast, we found that the small noncoding RNA *fnrS* is critical for these phenotypes. We were able to complement the Δ*fnr* dye and sulfide phenotypes with *fnrS* (Fig. 6B and C). While complementation was sufficient to rescue the Δ*fnr* phenotype, the *ΔfnrS::Kan^R^* carrying the empty plasmid did not show a loss-of-function phenotype at our usual sampling time of 24 h (Fig. 6B and C). We hypothesized that this is likely due to alternative regulators activating *cyuAP* and other L-Cysteine metabolism genes. Thus, we measured FD&C Red No. 40 degradation and sulfide production in the knockout strain over time. *fnrS::Kan^R^* demonstrated a delayed onset of dye degradation and lower sulfide production compared to *wt* (Fig. 6D and E). Furthermore, *fnrS* expression was induced significantly more in response to L-Cysteine in *wt* relative to Δ*fnr E. coli* (Fig. 6F).

## DISCUSSION

**Fig 7:**
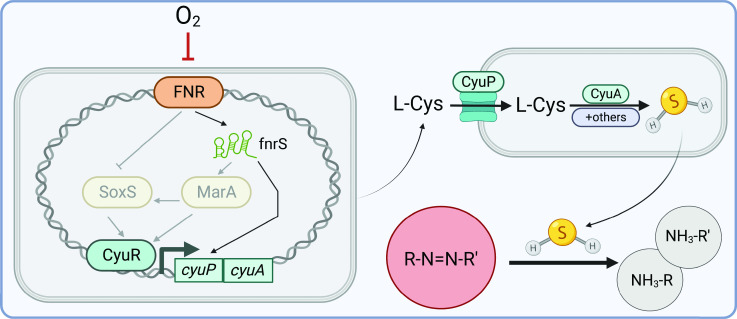
Working model describing how the host environment shapes gut bacterial azo reduction. Under anaerobic conditions, FNR activates the transcription of *fnrS,* a small noncoding regulatory RNA. *fnrS* in turn activates expression of the *cyuAP* operon. Alternatively, *fnrS* may upregulate the expression of *marA* which can drive expression of both *cyuR* and *soxS*. SoxS can also induce *cyuR* expression. CyuP transports L-Cysteine into the cell, where it is metabolized to H_2_S through the CyuA desulfidase. Released H_2_S can reduce the azo bond of azo compounds. Created with BioRender.com.

Our results provide mechanistic insight into the degradation of azo dyes by human gut bacteria. Surprisingly, the canonical azoreductase enzyme of *E. coli* is dispensable for whole-cell azoreductase activity. Instead, the shift to anaerobic growth is critical. We dissected a pathway through which oxygen is sensed by FNR, leading to upregulation of the *cyu* operon, increased uptake of L-Cysteine, enhanced production of H_2_S, and nonenzymatic azo bond reduction (Fig. 7).

These findings provide a cautionary tale for the ability to extrapolate results from biochemical studies of purified proteins to infer the metabolic activity of cells and complex microbial communities. Despite a rich literature on *E. coli* AzoR (24, 48, 73), we report that the *azoR* gene is dispensable for azo reduction by *E. coli* during anaerobic growth. These findings emphasize the importance of pairing biochemical and heterologous expression studies of enzyme function with methods for gene deletion in the original strain. Future work should extend this approach to the other bacterial species with biochemically characterized azoreductase enzymes (25, 29, 74, 75). For species where genetic tools do not exist (the vast majority of human gut bacteria), one could leverage natural strain-level variation to test the association between gene presence and sequence variation with metabolic activity (37).

The global regulator FNR is essential for *E. coli* azoreduction, highlighting the importance of environmental oxygen for the gut bacterial metabolism of drugs and excipients. Confirming the prior work on FNR in different growth conditions (42, 61), we confirmed that the FNR regulon is vast, including hundreds of genes involved in different aspects of anaerobic metabolism. Interestingly, efforts to utilize bacterial azoreduction to degrade dye runoff in water systems also require anaerobic conditions, suggesting that our findings may be generalizable to microbial communities outside of the human gut (23, 76, 77). Importantly, *E. coli* exhibits features of both aerobic and anaerobic growth within the GI tract (61, 78), depending upon its physical niche and host pathophysiology (79, 80). Our results suggest that inter-individual variations in the environment in which *E. coli* grows could have downstream consequences for its ability to act upon azo bond-containing compounds. More work is necessary to assess how the redox potential of the gut lumen and other environmental factors shape gut bacterial azo reduction in the context of host health and disease.

Surprisingly, FNR controls azoreductase potential through the reduction of azo bonds by H_2_S. Given that L-Cysteine is the major source of H_2_S for *E. coli*, these results provide an additional layer of environmental control for azoreduction. Differences in dietary protein, host proteoglycans, and/or L-Cysteine biosynthesis by other members of the gut microbiota could all potentially influence the activity of *E. coli* and its ability to act on azo bonds. Future studies leveraging gnotobiotic mice, defined dietary interventions, and isotope tracing could be informative in this regard.

Our results indicate that the L-Cysteine utilization regulator *cyuR*, among others, is under indirect FNR control, emphasizing the complex multi-level regulatory network contributing to bacterial azoreduction. The *cyu* operon is the main source for L-Cysteine import and metabolism by *E. coli* (63, 81). We also found that H_2_S production requires *cyuR.* Interestingly, the L-Cysteine uptake transporter, CyuP, was more important than the enzyme CyuA, suggesting that the import of L-Cysteine could provide substrate for additional pathways for H_2_S production. CyuA only functions anaerobically (63), consistent with our observations that azoreduction only occurs anaerobically and is regulated by FNR and CyuR. Moreover, the key role of L-Cysteine import suggests that amino acid availability from diet and/or host tissues could be critical for gut bacterial azoreduction. Future studies with mouse models and various dietary amino acid interventions could provide an opportunity to modulate microbiome azoreductase activity.

Deletion of both *cyuR* and *azoR* did not fully abolish *E. coli* azoreduction. Thus, additional pathways for L-Cysteine metabolism and/or as-of-yet undiscovered enzyme(s) may contribute to the metabolism of azo dyes by *E. coli*. In our initial screen, we found 21 deletion strains with a partial loss-of-function phenotype, all of which encode enzymes theoretically capable of azo reduction. Of these, four were also identified in our paired proteomic and transcriptomic data set (Table S6), which could be viable candidates for further biochemical characterization and/or complementation screens in the *Δfnr* background.

H_2_S production is widespread throughout gut bacterial species (82), suggesting that far more bacterial taxa are capable of azo reduction than previously appreciated. L-Cysteine can be made from a variety of different sources, thus methionine and other sulfur starting points could impact the gut microbiota’s H_2_S production capacity. Multiple *Desulfovibrio* species found in the GI tract can reduce sulfate to H_2_S (83 – 86). Thus, GI levels of H_2_S represent the net effect of multiple distinct pathways, which will be important to consider when translating these results into humans. As a first step, studies in gnotobiotic mice with defined microbial communities representing distinct assemblages of H_2_S producers would help to better understand the relative importance of each species for azo dye degradation under different dietary and host selection pressures.

There are multiple limitations of this study. We focused entirely on the model gut proteobacterium *E. coli* due to its genetic tractability and long history of molecular research; however, the generalizability of the pathway we described to other gut bacteria remains unclear. The development of genetic tools that permit similar mechanistic dissections into these more exotic gut bacteria will be essential for studying the conservation of enzymatic and nonenzymatic azo bond degradation in diverse gut bacterial species. The physiological relevance of our findings remains unclear, requiring more work in mice or other preclinical models. It will also be important to test the clinical relevance of these findings in the context of diseases intentionally treated with azo bond-containing drugs (e.g*.,* inflammatory bowel disease) or diseases such as cancer that could potentially be exacerbated by dietary exposures to azo dyes. Our results emphasize that in addition to metagenomics, it will be critical to measure key environmental factors in human clinical cohorts such as oxygen and amino acid levels.

Additionally, our results favor the hypothesis that FNR indirectly regulates *cyuR* and in turn *cyuAP*. While our data indicate that *fnrS* is responsible for the majority of this indirect regulation, these results do not address the combinatorial impact of all intermediate regulators. Ideally, a quadruple knockout of *marA*, *soxS*, *fnrS*, and *cyuR* would be necessary to confirm these are the only pathways leading toward *cyuAP* expression and thus H_2_S production. These results again highlight the complex regulation that underlies metabolic pathways and the importance of taking a nuanced approach to understanding not only gene product function but regulatory factors.

Despite these limitations, our results clearly emphasize the importance of environmental factors in controlling a clinically relevant metabolic activity of the human gut microbiome. These experiments are particularly timely given the rapid increase in food dye intake due to their incorporation in processed foods (87) and emerging evidence that they have unintended consequences for gut epithelial (18) and immune (22) cells. Continued progress toward dissecting the mechanisms responsible for this metabolic activity in *E. coli* and other experimentally tractable gut bacterial species is a key step toward predicting and controlling the location and extent of azo dye metabolism.

## MATERIALS AND METHODS

### Bacterial strains, media, and chemicals

Bacterial strains used in this study are listed in Table S7. *E. coli* was grown in lysogeny broth (LB) supplemented with 0.05% L-Cysteine under anaerobic conditions at 37°C unless otherwise noted. L-Cysteine was purchased from Sigma-Aldrich (St. Louis, MO).

### Assaying whole-cell *E. coli* depletion of azo compounds

Cultures were grown in LB medium supplemented with 250 µM of azo dye and 0.05% L-Cysteine. Azo dye stocks were prepared in DMSO at a concentration of 25 mM and used at a final concentration of 1% (v/v). Cultures were incubated at 37°C. All incubations were performed in a COY Laboratory Products Inc anaerobic chamber (Grass Lake, MI) with the following atmosphere: 5% H_2_, 20% CO_2_, and 75% N_2_. All reagents were equilibrated in the anaerobic chamber for at least 24 h before use. After 24 h of growth, samples were removed, spun down to remove cells, and analyzed for residual dye concentration by measuring absorbance at appropriate wavelengths. Wavelengths used: 450 nm (FD&C Red No. 40, FD&C Yellow No. 6, D&C Orange No. 4), 360 nm (balsalazide, sulfasalazine, olsalazine, phenazopyridine), 420 nm (D&C Brown No. 1), 500 nm (D&C Red No. 6, FD&C Red No. 4), 535 nm (D&C Red No. 33). Residual dye concentration was determined by creating a standard curve of dye concentrations in LB and measuring absorbance at appropriate wavelengths. Concentration was calculated using the equation of the fitted standard curve line.

### Clean deletion creation from Keio collection strains

Competent cells were created from cultures of the Keio collection knockouts for *azoR::Kan^R^* and *fnr::Kan^R^* knockouts. Cells were electroporated with vector pSIJ8 (88) and transformants were selected for on LB supplemented with 25 µg/mL ampicillin at 30°C. Removal of the kanamycin cassette at FRT sites was done on liquid culture of transformant using 50 mM L-rhamnose for 4 h, then patch streaked to identify loss of kanamycin resistance. Transformants were streak-purified two times, and then PCR was performed for confirmation of kanamycin cassette removal. Strains were cured of plasmid at 37°C and then patch-streaked to confirm loss of ampicillin resistance.

### Purified enzyme preparation

The ASKA collection (38) strain expressing *azoR* was streaked from glycerol stock on LB with 30 µg/mL chloramphenicol. A single colony was used to inoculate an overnight culture of 25 mL of LB with 30 µg/mL chloramphenicol aerobically at 37°C. The overnight was used to inoculate 1 L of LB with 30 µg/mL chloramphenicol at a 1:10 dilution factor. Sample was grown with shaking aerobically to an optical density of 0.5 at 600 nm. Expression of *azoR* was induced with isopropyl β-D-1-thiogalactopyranoside (IPTG) at 1 mM concentration for 3 h. Cells were then cooled on ice water for 10 min and centrifuged at 3,500 × g for 15 min, washed with ice cold PBS, spun again, then cell pellets were frozen overnight at −20°C. Pellets were thawed on ice, then resuspended in buffer A (50 mM HEPES, 300 mM NaCl, 10 mM imidazole, pH 7.5) and sonicated with the following program: 5 min, 4 s on, 4 s off, at 30% amplitude. Protease tablets (Sigma Aldrich) were crushed in buffer A and added to the sonicated lysate. Lysate was centrifuged for 20 min at 16,000 rpm. Supernatant was applied to a 5 mL nickel-nitrilotriacetic acid column (Qiagen, Valencia, CA) that had been equilibrated with buffer A at a rate of 1 mL/min. The column was washed with five column volumes of buffer A, then five column volumes of buffer B (50 mM HEPES, 300 mM NaCl, 20 mM imidazole, pH 7.5). Protein was eluted with buffer C (50 mM Hepes, 300 mM NaCl, 300 mM imidazole) and collected in 5 mL portions. Fractions were analyzed for protein content using a sodium dodecyl sulfate-polyacrylamide gel electrophoresis (SDS-PAGE) gel. Fractions containing proteins corresponding to AzoR molecular weight were pooled and dialyzed with 3 L of buffer D (50 mM Hepes, 300 mM NaCl, 15% glycerol by weight, in cold water, pH 7.5) overnight at 4°C. Protein concentration was determined using Nanodrop (Thermo Scientific).

### Purified enzyme experiments

AzoR reactions with azo dyes were carried out in 20 mM sodium phosphate buffer, 100 µM azo dye (unless otherwise noted), 2,000 µM NADH, and 20 µM FMN with varying concentrations of enzyme as appropriate. Reactions were activated by the addition of NADH. Dye concentration was monitored at absorbance 450 nm every 30 s after a linear shake of 15 s. All reactions were performed aerobically at 37°C.

### Keio collection screening for alternative azo reducers

Homologs to genes implicated in azoreduction by *S. oneidensis* MR-1 (40) were identified by first gene name and second gene function (tested or predicted) in *E. coli*. Other strains of interest were identified based on gene name and function. All strains were cross-referenced on EcoCyc (41). Strains were revived from glycerol stocks on LB agar with 25 µg/mL kanamycin. Single isolates were picked from agar plates and grown overnight in 1 mL of LB supplemented with 0.05% L-Cysteine in a 96-deep well plate under anaerobic conditions. For azoreduction assays, previously anaerobically equilibrated liquid media was supplemented with 250 µM FD&C Red No. 40 and 0.05% L-Cysteine. 1 mL of media was aliquoted to 2 mL deep 96-well plates and strains were inoculated at 1:100 from overnight cultures in triplicate. Plates were sealed with TempPlate Sealing Foil (USA Scientific, Cat #2923–0110) and allowed to grow for 24 h. After 24 h, cells were spun down and 100 µL of supernatant was used to measure dye absorbance at 450 nm. Proportion of dye remaining was calculated by dividing the absorbance of each well by the average absorbance of uninoculated control wells. Where standard curves were used, a 1:2 dilution series of FD&C Red No. 40 was made in LB with 0.05% L-Cysteine in triplicate and dye absorbance measured at 450 nm. Concentrations were calculated using the equation of the standard curve line in Excel.

### Validation of Keio collection knockouts

To ensure the knockouts used from the Keio collection were indeed the annotated gene, primers were designed up- and down-stream of the gene of interest. PCR and Sanger sequencing of the kanamycin insert and surrounding regions were performed to ensure the expected band size and gene alignment. Primers are listed in Table S8.

### Complement strain construction and assay

Complement strains were made by preparing competent cells for the background strain of interest. Plasmids were prepared by growing up overnight cultures from the ASKA strain of interest, then using Qiagen Plasmid Mini Kit (Qiagen; Cat #12125) to extract the pCA24N plasmid with the gene of interest. The plasmid preparation was desalted using a membrane filter on water, then electroporated to the background strain of interest. Correct insertion was ensured by growth of the transformant on chloramphenicol. A mini prep was also made from the transformant, and PCR and Sanger sequencing done to ensure the expected gene insertion. The same procedure was used to introduce the pBD-pLac and pBD-pLac-fnrS into strains of interest.

### RNA sequencing and proteomics sample growth and exposure

100 mL of overnight cultures of both *wt* and Δ*fnr E. coli* were grown in LB supplemented with 0.05% L-Cysteine under anaerobic conditions. Overnight culture OD_600 nm_ was measured for both cultures and normalized to a starting OD_600 nm_ of 0.05 in 100 mL of LB with 0.05% L-Cysteine in 12 replicates for each strain. OD_600 nm_ was monitored until mid-exponential phase for each strain (previously determined). At mid-exponential three replicates for each strain were treated with a final concentration of 250 µM FD&C Red No. 40 in DMSO, or 1% DMSO. After 45 min of exposure, 15 mL of the sample was removed for RNA extraction, while the remaining volume was used for proteomics. Both volumes of samples were spun down to pellet cells, supernatant removed, and cell pellets were flash frozen in liquid nitrogen then stored at −80°C. The remaining samples were allowed to reach stationary phase, then they were treated with a final concentration of 250 µM FD&C Red No. 40 in DMSO, or 1% DMSO. Samples were processed the same way as for mid-exponential.

### RNA sequencing sample preparation and analysis

1 µL of TRI Reagent (Sigma Aldrich catalog number T9424) was added to bacterial pellets and incubated for 10 min, samples were then transferred to 2 mL Lysing Matrix E tubes (MP Biomecials, catalog number 116914050). Cells were lysed for 5 min in the bead beater at room temperature. 200 µL of chloroform was added. Samples were vortexed for 15 s and incubated at room temperature for 10 min. Samples were then centrifuged at 16,000 × g for 15 min at 4°C. 500 µL of the upper aqueous phase was transferred to a new tube, 500 µL of 100% ethanol was added, and vortexed to mix. Mixture was transferred to a spin column (PureLink RNA Mini Kit; Life Technologies; catalog number 12183025) and centrifuged at ≥12,000 × g for 30  s, discarding flow-through, until all the material had been added to the column. To the spin column, 350  µL Wash Buffer I (PureLink RNA Mini Kit; Life Technologies; catalog number 12183025) was added, then the column was centrifuged at ≥12,000 × g for 30  s, discarding flow-through. 80 µL of PureLink DNase mix was added to the column and incubated at room temperature for 15 min. 350 µL of Wash Buffer I (Purelink RNA mini kit) was added, and the column spun at >12,000 × g for 30 s. The column was transferred to a new collection tube, and 500  µL Wash Buffer II was added, followed by centrifugation at ≥12,000 × g for 30  s, discarding flow-through. The column was centrifuged at ≥12,000 × g for 60  s and dried, then moved to a collection tube. 50  µL RNase-free water was added, and the column was incubated at room temperature for 1  min. Finally, the column was centrifuged for 1  min at ≥12,000 × g, retaining the flow-through, which contained total RNA.

Samples were DNase treated again using TURBO-DNase (Ambion; ThermoFisher catalog number AM2238) and incubated at 37°C for 30 min. RNA Ampure XP Beads were used to clean up the reactions. 1.8 volumes of the RNA Ampure XP beads were added to 1 vol of RNA sample and allowed to sit for 5 min at room temperature. Tubes were placed on a magnetic stand until the liquid cleared. Then liquid was removed, and beads washed with 200 µL of 100% ethanol. Samples were incubated for 30 s, then removed. This was repeated twice. Samples were allowed to dry for 5 min, removed from the magnetic stand, and 30 µL of RNase-free water was added. This was incubated for 2 min at room temperature then placed back on the magnetic rack where liquid was collected after turning clear.

rRNA was removed from total RNA using Ribominus Transcriptome Isolation Kit for Bacteria and Yeast (Invitrogen; catalog number K155004, LOT: 2116711), following manufacturer’s protocol. RNA fragmentation, cDNA synthesis, and library preparation were performed using the NEBNext Ultra RNA library Prep Kit for Illumina and NEBNext Multiplex Oligos for Illumina (Dual Index Primers) (Ipswich, MA). Samples were dual-end sequenced (2 × 75 bp) using the NextSeq Mid Output platform (Table S2). Reads were mapped to the *E. coli* K12 BW25113 genome sequence (NCBI Reference Sequence: GCA_000750555.1) using Bowtie2 (89), and HTSeq (90) was used to count the number of reads to *E. coli* genes. Differential gene expression was analyzed using limma (91). Differentially expressed genes were defined as transcripts exhibiting an absolute log_2_ fold change ≥1 and p_adj_ <0.1. Detection of expression was based on limma’s default filtering parameters in the “filter genes by expression level” function.

### Proteomics sample preparation

Cell pellets were washed three times with 5 mL of cold PBS and resuspended in 1 mL, transferred to 2 mL Lysing Matrix E tubes (MP Biomecials, catalog number 116914050) and lysed via bead beating for 1 min, then resting tubes on ice for 2 min. This was repeated twice. Samples were centrifuged for 15 min at 4°C at 16,000 × g. A BCA assay was performed to quantify protein concentration in the lysate supernatant. Urea was added to the samples to a target concentration of 8 M followed by an appropriate volume of dithiothreitol to obtain a 5 mM concentration. Samples were then incubated at 60°C for 30 min. While samples were incubating, the trypsin was preactivated for 10 min at 37°C. Samples were diluted 10-fold with 100 mM NH_4_HCO_3_. 1 M CaCl_2_ was added at an appropriate volume to create a final sample concentration of 1 mM CaCl_2_. Samples were then digested for 3 h with trypsin at 37°C at a concentration of 1 µg trypsin/50 µg protein. Samples were snap frozen.

Samples were cleaned with a C_18_ column on a vacuum manifold. The column was conditioned with 3 mL of methanol, then rinsed with 2 mL of 0.1% TFA acidified water. Sample was run though column, then column was rinsed with 4 mL of 95:5 H_2_O:acetonitrile (ACN), 0.1% trifluoroacetic acid (TFA). The column was allowed to go to dryness, then eluted slowly to dryness with 1 mL of 80:20 ACN:H_2_O, 0.1% TFA into a collection tube. Samples were concentrated in the speed-vac to a volume of approximately 50–100 μL. Protein quantification was done using a BCA test, then samples were stored at −80°C until analysis.

### Proteomics

A Waters nano-Acquity M-Class dual pumping UPLC system (Milford, MA) was configured for online trapping of a 5 µL injection at 5 µL/min with reverse-flow elution into the analytical column at 300 nL/min. The trapping column was packed in-house (PNNL) using 360 µm o.d. fused silica (Polymicro Technologies Inc., Phoenix, AZ) with 5 mm Kasil frits for media retention and contained Jupiter C_18_ media (Phenomenex, Torrence, CA) in 5 µm particle size for the trapping column (150 µm i.d.  × 4 cm long) and 3 µm particle size for the analytical column (75 µm i.d.  × 70 cm long). Mobile phases consisted of (A) water with 0.1% formic acid and (B) acetonitrile with 0.1% formic acid. The following gradient profile was performed (min, %B): 0, 1; 2, 8; 25, 12; 85, 35; 105, 55; 110, 95; 115, 95; 117, 50; 119, 95; 121, 95; 123, 1.

MS analysis was performed using a Velos Orbitrap Elite mass spectrometer (Thermo Scientific, San Jose, CA) outfitted with an in-house made nano-electrospray ionization interface. Electrospray emitters were prepared using 150 µm o.d.  × 20 µm i.d. chemically etched fused silica (92). The ion transfer tube temperature and spray voltage were 325°C and 2.2 kV, respectively. Data were collected for 100 min following a 20 min delay from sample injection. FT-MS spectra were acquired from 400 to 2000* m/z* at a resolution of 35 k (AGC target 3e6) and while the top 12 FT-HCD-MS/MS spectra were acquired in data-dependent mode with an isolation window of 2.0* m*/*z* and at a resolution of 17.5 k (AGC target 1e5) using a normalized collision energy of 30 s and a 30 s exclusion time. Extracted MS data were run through the MS-GF+ database and filtered by a FDR of 0.01 using the MS-GF+ generated decoy database. Differential peptide abundance was analyzed using DEP (93) and limma (91) using a cutoff of an absolute log_2_ fold change ≥1 and p_adj_ < 0.1.

### Regulator enrichment analysis

Features found significantly differentially expressed (log_2_ fold change > |1|, *p_adj_* < 0.1) in both the proteomics and transcriptomics data sets were imported to EcoCyc (41) for enrichement analysis. The gene list was analyzed using the ”Genes enriched for transcriptional/translational regulators (direct only)”. The following settings were used: *p_adj_* < 0.1, Fisher Exact Statistic, Benjamini-Hochberg Correction). Proportion of regulon represented was determined using the list of genes “directly regulated by gene” feature of EcoCyc for total regulon size. Then, the number of genes regulated by each regulator was divided by number of total genes regulated by that particular regulator.

### Hydrogen sulfide quantification

H_2_S was measured using the Cline reaction (94) with modifications. Samples were put into a zinc acetate buffer (16.7 mM) on ice at a ratio of 1:3 sample:buffer. Then 180 µL of zinc acetate solution was transferred and mixed with 20 µL of line reagent (2 g of *N,N*-dimethyl-*p*-phenylenediamine sulfate with 3 g of FeCl_3_ in 500 mL of cold 50% v/v HCl). The reaction was allowed to proceed for 20–30 min at room temperature in the dark. Then absorbances were measured at 670 nm. A standard curve of sodium sulfide was created in zinc acetate and reacted with Cline reagents for sulfide concentration calculations.

### RT-qPCR assays

Samples were grown under anaerobic conditions with 250 µM FD&C Red No. 40. At mid-exponential phase, 4.2 mM L-Cysteine was added to cultures for 15 min. Then samples were removed from anaerobic conditions, centrifuged for 5  min at ≥12,000 × g, supernatant removed, and pellets were frozen at −80°C. Samples were thawed and RNA extracted as previously described for RNA sequencing. RT-qPCR was done using RioRad’s 1-step Quantitative Reverse Transcription PCR kit (Bio-Rad, Hercules, CA, catalog number 1725150) according to kit protocol.

To analyze the differences between *wt* and *Δfnr* samples, fold change of sample ΔC_t_ minus the maximum ΔC_t_ for each gene across all samples in *wt* or *Δfnr E. coli* was calculated. Within each replicate the Ct of the housekeeping gene was subtracted from the C_t_ of the gene of interest (ΔC_t_). The maximum ΔC_t_ across samples from both strains was subtracted from all samples to give ΔΔC_t_. Fold change was calculated as 2^(-ΔΔC_t_). To analyze the impact of L-Cysteine on gene expression, fold change was calculated using expression of the sample with L-Cysteine compared to the vehicle. Within each replicate the C_t_ of the housekeeping gene was subtracted from the C_t_ of the gene of interest (ΔC_t_). The ΔC_t_ from the paired replicate control (vehicle exposure) samples was subtracted from the exposed sample ΔC_t_ to give ΔΔC_t_. Fold change was calculated as 2^(-ΔΔC_t_).

### Double gene knockout creation

P1 lysates were generated of each strain of interest carrying the kanamycin resistance cassette (Keio collection) adapting methods from previously described techniques (95). Briefly, 150 µL of overnight culture in LB supplemented with 12.5 µg/mL kanamycin was mixed with 1 to 25 µL of P1 phage (previously propagated from ATCC on MG1655). This mixture was incubated for 10 min at 37°C to aid absorption, added to 3 mL of 0.7% agar, and overlaid on prewarmed LB agar supplemented with 25 µg/mL kanamycin and 10 mM MgSO_4_. Plates were incubated overnight at 37°C, and phage was harvested by adding 5 mL of SM buffer, incubating at room temperature for 10 min, and breaking the top agar for phage harvest. The mixture was briefly centrifuged to pellet agar, then supernatant was passed through a 100 µm cell straining, then 0.45 µm syringe filter. Lysates were stored at 4°C.

A clean deletion of the recipient strain of interest was created using the earlier described method with pSIJ8 (88). To transduce the clean recipient strain, 1 mL of an overnight culture of recipient strain was pelleted and resuspended in ⅓ volume of LB 10 mM MgSO_4_, 5 mM CaCl_2_. 100 µL of cells were mixed with 1 μL to 10 µL of P1 lysate and incubated for 60 min at 37°C. 200 µL of 1M sodium citrate was added with 1 mL of LB to minimize secondary infection. This mixture was incubated at 37°C for 2 h, then plated on LB supplemented with 10 mM sodium citrate and 25 µg/mL kanamycin to select for transductants. Transduction was confirmed using PCR and Sanger sequencing. Azoreduction and sulfide activity were tested as described earlier.

### Statistical analysis

For multiple comparisons of data, an ordinary one-way ANOVA test with Dunnet’s correction was chosen, unless otherwise specified. For the targeted knockout screen, a 95% CI of averages from two experiments was calculated using GraphPad Prism v9. Michaelis-Menten curves were created in GraphPad Prism v9. For proteomics and transcriptomics analysis, R package limma was used to determine the fold change and adjusted *P* values. Feature count differences and Mann-Whitney significance testing were done in R. For the regulator enrichment analysis, EcoCyc’s enrichment analysis for “Genes enriched for transcriptional/translational regulators (direct only)” was used with a Fisher’s exact test with Benjamini-Hochberg correction.

## Data Availability

RNA-seq data are available through the NCBI Gene Expression Omnibus (GEO) online data repository under accession number GSE235465. Proteomics data are available through ProteomeXchange.org, accession number PXD043589. All other data are provided in the supplemental tables or available upon request.

## SUPPLEMENTAL MATERIAL

Fig. S1 mbio.01573-23-s0001.eps Validation of bacterial strains.

Fig. S2 mbio.01573-23-s0002.eps
*E. coli* AzoR is sufficient to deplete azo dyes.

Fig. S3 mbio.01573-23-s0003.eps
*fnr* is necessary for the depletion of multiple azo dyes

Fig. S4 mbio.01573-23-s0004.eps Dye depletion corresponds to the growth phase of wt *E. coli*.

Fig. S5 mbio.01573-23-s0005.eps
*ydeN* is not required for azo dye depletion

Fig. S6 mbio.01573-23-s0006.eps Key transcripts and proteins in Δ*fnr E. coli* relative to wild-type controls.

Fig. S7 mbio.01573-23-s0007.eps Regulation of *cyuR* and its operon.

Supplemental Tables mbio.01573-23-s0008.xlsx Tables S1 to S8.

ASM does not own the copyrights to Supplemental Material that may be linked to, or accessed through, an article. The authors have granted ASM a non-exclusive, world-wide license to publish the Supplemental Material files. Please contact the corresponding author directly for reuse.

## References

[1] Spanogiannopoulos P , Bess EN , Carmody RN , Turnbaugh PJ . 2016. The microbial pharmacists within us: a metagenomic view of xenobiotic metabolism. Nat Rev Microbiol 14:273–287. doi:10.1038/nrmicro.2016.17 26972811 PMC5243131

[2] Collins SL , Patterson AD . 2020. The gut microbiome: an orchestrator of xenobiotic metabolism. Acta Pharm Sin B 10:19–32. doi:10.1016/j.apsb.2019.12.001 31998605 PMC6984741

[3] Clarke G , Sandhu KV , Griffin BT , Dinan TG , Cryan JF , Hyland NP . 2019. Gut reactions: breaking down xenobiotic-microbiome interactions. Pharmacol Rev 71:198–224. doi:10.1124/pr.118.015768 30890566

[4] Yee Yip L , Chun Yong Chan E . 2015. Special section on drug metabolism and the microbiome—minireview investigation of host–gut microbiota modulation of therapeutic outcome. Drug Metab Dispos 43:1619–1631. doi:10.1124/dmd.115.063750 25979259

[5] Koppel N , Maini Rekdal V , Balskus EP . 2017. Chemical transformation of xenobiotics by the human gut microbiota. Science 356:eaag2770. doi:10.1126/science.aag2770 28642381 PMC5534341

[6] Nayak RR , Turnbaugh PJ . 2016. Mirror, mirror on the wall: which microbiomes will help heal them all? BMC Med 14:72. doi:10.1186/s12916-016-0622-6 27146150 PMC4857263

[7] Peppercorn MA . 1984. Sulfasalazine. pharmacology, clinical use, toxicity, and related new drug development. Ann Intern Med 101:377–386. doi:10.7326/0003-4819-101-3-377 6147110

[8] Center for Food Safety, Nutrition A, US Food and Drug Administration . Color additives history. FDA. Available from: https://www.fda.gov/industry/color-additives/color-additives-history. Retrieved 2424 JunJune 2022. Accessed , 2424 JunJune 2022

[9] Misal SA , Gawai KR . 2018. Azoreductase: a key player of xenobiotic metabolism. Bioresour Bioprocess 5. doi:10.1186/s40643-018-0206-8

[10] Chung KT , Fulk GE , Egan M . 1978. Reduction of azo dyes by intestinal anaerobes. Appl Environ Microbiol 35:558–562. doi:10.1128/aem.35.3.558-562.1978 25047 PMC242879

[11] Chung KT , Stevens SE , Cerniglia CE . 1992. The reduction of azo dyes by the intestinal microflora. Crit Rev Microbiol 18:175–190. doi:10.3109/10408419209114557 1554423

[12] Wang R-F , Chen H , Paine DD , Cerniglia CE . 2004. Microarray method to monitor 40 intestinal bacterial species in the study of azo dye reduction. Biosens Bioelectron 20:699–705. doi:10.1016/j.bios.2004.04.011 15522584 PMC5875181

[13] Zahran SA , Ali-Tammam M , Hashem AM , Aziz RK , Ali AE . 2019. Azoreductase activity of dye-decolorizing bacteria isolated from the human gut microbiota. Sci Rep 9:5508. doi:10.1038/s41598-019-41894-8 30940826 PMC6445285

[14] Ryan A . 2017. Azoreductases in drug metabolism. Br J Pharmacol 174:2161–2173. doi:10.1111/bph.13571 27487252 PMC5481658

[15] Gingell R , Bridges JW , Williams RT . 1971. The role of the gut flora in the metabolism of prontosil and neoprontosil in the rat. Xenobiotica 1:143–156. doi:10.3109/00498257109044386 5173017

[16] Sousa T , Yadav V , Zann V , Borde A , Abrahamsson B , Basit AW . 2014. On the colonic bacterial metabolism of azo-bonded prodrugs of 5-aminosalicylic acid. J Pharm Sci 103:3171–3175. doi:10.1002/jps.24103 25091594

[17] Stevens LJ , Kuczek T , Burgess JR , Stochelski MA , Arnold LE , Galland L . 2013. Mechanisms of behavioral, atopic, and other reactions to artificial food colors in children. Nutr Rev 71:268–281. doi:10.1111/nure.12023 23590704

[18] Zou L , Spanogiannopoulos P , Pieper LM , Chien H-C , Cai W , Khuri N , Pottel J , Vora B , Ni Z , Tsakalozou E , Zhang W , Shoichet BK , Giacomini KM , Turnbaugh PJ . 2020. Bacterial metabolism rescues the inhibition of intestinal drug absorption by food and drug additives. Proc Natl Acad Sci U S A 117:16009–16018. doi:10.1073/pnas.1920483117 32571913 PMC7355017

[19] Chen H . 2012. Toxicological significance of azo dye metabolism by human intestinal microbiota. Front Biosci E4:568–586. doi:10.2741/e400 PMC5870118 22201895

[20] Yahagi T , Degawa M , Seino Y , Matsushima T , Nagao M . 1975. Mutagenicity of carcinogenic azo dyes and their derivatives. Cancer Lett 1:91–96. doi:10.1016/s0304-3835(75)95563-9 828073

[21] Chung K-T . 1983. The significance of azo-reduction in the mutagenesis and carcinogenesis of azo dyes. Mutat Res 114:269–281. doi:10.1016/0165-1110(83)90035-0 6339890

[22] He Z , Chen L , Catalan-Dibene J , Bongers G , Faith JJ , Suebsuwong C , DeVita RJ , Shen Z , Fox JG , Lafaille JJ , Furtado GC , Lira SA . 2021. Food colorants metabolized by commensal bacteria promote colitis in mice with dysregulated expression of interleukin-23. Cell Metab 33:1358–1371. doi:10.1016/j.cmet.2021.04.015 33989521 PMC8266754

[23] Sandhya S . 2010. Biodegradation of azo dyes under anaerobic condition: role of azoreductase, p 39–57. In Atacag Erkurt H (ed), Biodegradation of azo dyes. Springer, Berlin Heidelberg. doi:10.1007/978-3-642-11847-0

[24] Nakanishi M , Yatome C , Ishida N , Kitade Y . 2001. Putative ACP phosphodiesterase gene (acpD) encodes an azoreductase. J Biol Chem 276:46394–46399. doi:10.1074/jbc.M104483200 11583992

[25] Nachiyar CV , Rajakumar GS . 2005. Purification and characterization of an oxygen insensitive azoreductase from Pseudomonas aeruginosa. Enzyme Microb Technol 36:503–509. doi:10.1016/j.enzmictec.2004.11.015

[26] Wang C-J , Hagemeier C , Rahman N , Lowe E , Noble M , Coughtrie M , Sim E , Westwood I . 2007. Molecular cloning, characterisation and ligand-bound structure of an azoreductase from Pseudomonas aeruginosa. J Mol Biol 373:1213–1228. doi:10.1016/j.jmb.2007.08.048 17904577

[27] Ryan A , Kaplan E , Nebel J-C , Polycarpou E , Crescente V , Lowe E , Preston GM , Sim E . 2014. Identification of NAD(P)H quinone oxidoreductase activity in azoreductases from P. aeruginosa: azoreductases and NAD(P)H quinone oxidoreductases belong to the same FMN-dependent superfamily of enzymes. PLoS One 9:e98551. doi:10.1371/journal.pone.0098551 24915188 PMC4051601

[28] Crescente V , Holland SM , Kashyap S , Polycarpou E , Sim E , Ryan A . 2016. Identification of novel members of the bacterial azoreductase family in Pseudomonas aeruginosa. Biochem J 473:549–558. doi:10.1042/BJ20150856 26621870

[29] Chen H , Wang R-F , Cerniglia CE . 2004. Molecular cloning, overexpression, purification, and characterization of an aerobic FMN-dependent azoreductase from Enterococcus faecalis. Protein Expr Purif 34:302–310. doi:10.1016/j.pep.2003.12.016 15003265 PMC5875116

[30] Liu Z-J , Chen H , Shaw N , Hopper SL , Chen L , Chen S , Cerniglia CE , Wang B-C . 2007. Crystal structure of an aerobic FMN-dependent azoreductase (azoa) from Enterococcus faecalis. Arch Biochem Biophys 463:68–77. doi:10.1016/j.abb.2007.03.003 17428434

[31] Chalansonnet V , Mercier C , Orenga S , Gilbert C . 2017. Identification of Enterococcus faecalis enzymes with azoreductases and/or nitroreductase activity. BMC Microbiol 17:126. doi:10.1186/s12866-017-1033-3 28545445 PMC5445473

[32] Morrison JM , John GH . 2013. The non-enzymatic reduction of azo dyes by flavin and nicotinamide cofactors under varying conditions. Anaerobe 23:87–96. doi:10.1016/j.anaerobe.2013.07.005 23891960

[33] Dai Q , Zhang S , Liu H , Huang J , Li L . 2020. Sulfide-mediated azo dye degradation and microbial community analysis in a single-chamber air cathode microbial fuel cell. Bioelectrochemistry 131:107349. doi:10.1016/j.bioelechem.2019.107349 31476657

[34] Wolfson SJ , Hitchings R , Peregrina K , Cohen Z , Khan S , Yilmaz T , Malena M , Goluch ED , Augenlicht L , Kelly L . 2022. Bacterial hydrogen sulfide drives cryptic redox chemistry in gut microbial communities. Nat Metab 4:1260–1270. doi:10.1038/s42255-022-00656-z 36266544 PMC11328334

[35] Haiser HJ , Turnbaugh PJ . 2013. Developing a metagenomic view of xenobiotic metabolism. Pharmacol Res 69:21–31. doi:10.1016/j.phrs.2012.07.009 22902524 PMC3526672

[36] Zimmermann M , Zimmermann-Kogadeeva M , Wegmann R , Goodman AL . 2019. Mapping human microbiome drug metabolism by gut bacteria and their genes. Nature 570:462–467. doi:10.1038/s41586-019-1291-3 31158845 PMC6597290

[37] Bisanz JE , Soto-Perez P , Noecker C , Aksenov AA , Lam KN , Kenney GE , Bess EN , Haiser HJ , Kyaw TS , Yu FB , Rekdal VM , Ha CWY , Devkota S , Balskus EP , Dorrestein PC , Allen-Vercoe E , Turnbaugh PJ . 2020. A genomic toolkit for the mechanistic dissection of intractable human gut bacteria. Cell Host Microbe 27:1001–1013. doi:10.1016/j.chom.2020.04.006 32348781 PMC7292766

[38] Kitagawa M , Ara T , Arifuzzaman M , Ioka-Nakamichi T , Inamoto E , Toyonaga H , Mori H . 2005. Complete set of ORF clones of Escherichia coli ASKA library (A complete set of E. coli K-12 ORF archive): unique resources for biological research. DNA Res 12:291–299. doi:10.1093/dnares/dsi012 16769691

[39] Zhang H-K , Lu H , Wang J , Liu G-F , Zhou J-T , Xu M-Y . 2013. Global transcriptome analysis of Escherichia coli exposed to immobilized anthraquinone-2-sulfonate and azo dye under anaerobic conditions. Appl Microbiol Biotechnol 97:6895–6905. doi:10.1007/s00253-013-5066-8 23820558

[40] Brigé A , Motte B , Borloo J , Buysschaert G , Devreese B , Van Beeumen JJ . 2008. Bacterial decolorization of textile dyes is an extracellular process requiring a multicomponent electron transfer pathway. Microb Biotechnol 1:40–52. doi:10.1111/j.1751-7915.2007.00005.x 21261820 PMC3864430

[41] Karp PD , Ong WK , Paley S , Billington R , Caspi R , Fulcher C , Kothari A , Krummenacker M , Latendresse M , Midford PE , Subhraveti P , Gama-Castro S , Muñiz-Rascado L , Bonavides-Martinez C , Santos-Zavaleta A , Mackie A , Collado-Vides J , Keseler IM , Paulsen I . 2018. The EcoCyc database. EcoSal Plus 8. doi:10.1128/ecosalplus.ESP-0006-2018 PMC6504970 30406744

[42] Unden G , Trageser M . 1991. Oxygen regulated gene expression in Escherichia coli: control of anaerobic respiration by the FNR protein. Antonie Van Leeuwenhoek 59:65–76. doi:10.1007/BF00445650 1854188

[43] Spiro S , Guest JR . 1990. FNR and its role in oxygen-regulated gene expression in Escherichia coli. FEMS Microbiol Lett 75:399–428. doi:10.1111/j.1574-6968.1990.tb04109.x 2248796

[44] Myers KS , Yan H , Ong IM , Chung D , Liang K , Tran F , Keleş S , Landick R , Kiley PJ . 2013. Genome-scale analysis of Escherichia coli FNR reveals complex features of transcription factor binding. PLoS Genet 9:e1003565. doi:10.1371/journal.pgen.1003565 23818864 PMC3688515

[45] Srinivasan S , Venkatesh KV . 2014. Steady state analysis of the genetic regulatory network incorporating underlying molecular mechanisms for anaerobic metabolism in Escherichia coli. Mol Biosyst 10:562–575. doi:10.1039/c3mb70483a 24402032

[46] Kang Y , Weber KD , Qiu Y , Kiley PJ , Blattner FR . 2005. Genome-wide expression analysis indicates that FNR of Escherichia coli K-12 regulates a large number of genes of unknown function. J Bacteriol 187:1135–1160. doi:10.1128/JB.187.3.1135-1160.2005 15659690 PMC545700

[47] Gunsalus RP , Park SJ . 1994. Aerobic-anaerobic gene regulation in Escherichia coli: control by the arcab and Fnr regulons. Res Microbiol 145:437–450. doi:10.1016/0923-2508(94)90092-2 7855430

[48] Ito K , Nakanishi M , Lee W-C , Sasaki H , Zenno S , Saigo K , Kitade Y , Tanokura M . 2006. Three-dimensional structure of AzoR from Escherichia coli. An oxidereductase conserved in microorganisms. J Biol Chem 281:20567–20576. doi:10.1074/jbc.M513345200 16684776

[49] Blake T , Barnard A , Busby SJW , Green J . 2002. Transcription activation by FNR: evidence for a functional activating region 2. J Bacteriol 184:5855–5861. doi:10.1128/JB.184.21.5855-5861.2002 12374818 PMC135384

[50] Bilous PT , Weiner JH . 1985. Dimethyl sulfoxide reductase activity by anaerobically grown Escherichia coli HB101. J Bacteriol 162:1151–1155. doi:10.1128/jb.162.3.1151-1155.1985 3888958 PMC215896

[51] Unden G , Bongaerts J . 1997. Alternative respiratory pathways of Escherichia coli: energetics and transcriptional regulation in response to electron acceptors. Biochim Biophys Acta 1320:217–234. doi:10.1016/s0005-2728(97)00034-0 9230919

[52] Kim S , Pevzner PA . 2014. MS-GF+ makes progress towards a universal database search tool for proteomics. Nat Commun 5:1–10. doi:10.1038/ncomms6277 PMC5036525 25358478

[53] Caglar MU , Houser JR , Barnhart CS , Boutz DR , Carroll SM , Dasgupta A , Lenoir WF , Smith BL , Sridhara V , Sydykova DK , Vander Wood D , Marx CJ , Marcotte EM , Barrick JE , Wilke CO . 2017. The E. coli molecular phenotype under different growth conditions. Sci Rep 7:45303. doi:10.1038/srep45303 28417974 PMC5394689

[54] Makinoshima H , Aizawa S-I , Hayashi H , Miki T , Nishimura A , Ishihama A . 2003. Growth phase-coupled alterations in cell structure and function of Escherichia coli. J Bacteriol 185:1338–1345. doi:10.1128/JB.185.4.1338-1345.2003 12562804 PMC142870

[55] Kobayashi A , Hirakawa H , Hirata T , Nishino K , Yamaguchi A . 2006. Growth phase-dependent expression of drug exporters in Escherichia coli and its contribution to drug tolerance. J Bacteriol 188:5693–5703. doi:10.1128/JB.00217-06 16885437 PMC1540079

[56] Keseler IM , Collado-Vides J , Santos-Zavaleta A , Peralta-Gil M , Gama-Castro S , Muñiz-Rascado L , Bonavides-Martinez C , Paley S , Krummenacker M , Altman T , Kaipa P , Spaulding A , Pacheco J , Latendresse M , Fulcher C , Sarker M , Shearer AG , Mackie A , Paulsen I , Gunsalus RP , Karp PD . 2011. EcoCyc: a comprehensive database of Escherichia coli biology. Nucleic Acids Res 39:D583–D590. doi:10.1093/nar/gkq1143 21097882 PMC3013716

[57] Keseler IM , Gama-Castro S , Mackie A , Billington R , Bonavides-Martínez C , Caspi R , Kothari A , Krummenacker M , Midford PE , Muñiz-Rascado L , Ong WK , Paley S , Santos-Zavaleta A , Subhraveti P , Tierrafría VH , Wolfe AJ , Collado-Vides J , Paulsen IT , Karp PD . 2021. The EcoCyc database in 2021. Front Microbiol 12:711077. doi:10.3389/fmicb.2021.711077 34394059 PMC8357350

[58] Inada T , Takahashi H , Mizuno T , Aiba H . 1996. Down regulation of cAMP production by cAMP receptor protein in Escherichia coli: an assessment of the contributions of transcriptional and posttranscriptional control of adenylate cyclase. Mol Gen Genet 253:198–204. doi:10.1007/s004380050313 9003304

[59] Zheng D , Constantinidou C , Hobman JL , Minchin SD . 2004. Identification of the CRP regulon using in vitro and in vivo transcriptional profiling. Nucleic Acids Res 32:5874–5893. doi:10.1093/nar/gkh908 15520470 PMC528793

[60] Shimada T , Fujita N , Yamamoto K , Ishihama A . 2011. Novel roles of cAMP receptor protein (CRP) in regulation of transport and metabolism of carbon sources. PLoS One 6:e20081. doi:10.1371/journal.pone.0020081 21673794 PMC3105977

[61] Kargeti M , Venkatesh KV . 2017. The effect of global transcriptional regulators on the anaerobic fermentative metabolism of Escherichia coli. Mol Biosyst 13:1388–1398. doi:10.1039/c6mb00721j 28573283

[62] Loddeke M , Schneider B , Oguri T , Mehta I , Xuan Z , Reitzer L . 2017. Anaerobic cysteine degradation and potential metabolic coordination in Salmonella enterica and Escherichia coli. J Bacteriol 199:e00117-17. doi:10.1128/JB.00117-17 28607157 PMC5527379

[63] Zhou Y , Imlay JA , Hammer ND , Heran Darwin K . 2022. Escherichia coli uses a dedicated importer and desulfidase to ferment cysteine. mBio 13:e0296521. doi:10.1128/mbio.02965-21 35377168 PMC9040844

[64] Shimada T , Tanaka K , Ishihama A . 2016. Transcription factor DecR (YbaO) controls detoxification of L-cysteine in Escherichia coli. Microbiology (Reading) 162:1698–1707. doi:10.1099/mic.0.000337 27435271

[65] Zhao H-Q , Huang S-Q , Xu W-Q , Wang Y-R , Wang Y-X , He C-S , Mu Y . 2019. Undiscovered mechanism for pyrogenic carbonaceous matter-mediated abiotic transformation of azo dyes by sulfide. Environ Sci Technol 53:4397–4405. doi:10.1021/acs.est.8b06692 30908036

[66] Li K , Xin Y , Xuan G , Zhao R , Liu H , Xia Y , Xun L . 2019. Escherichia coli uses separate enzymes to produce H_2_S and reactive sulfane sulfur from L-cysteine. Front. Microbiol 10:298. doi:10.3389/fmicb.2019.00298 30873134 PMC6401616

[67] Hungate RE . 1950. The anaerobic mesophilic cellulolytic bacteria. Bacteriol Rev 14:1–49. doi:10.1128/br.14.1.1-49.1950 15420122 PMC440953

[68] Fukushima RS , Weimer PJ , Kunz DA . 2003. Use of photocatalytic reduction to hasten preparation of culture media for saccharolytic Clostridium species. Braz J Microbiol 34:22–26. doi:10.1590/S1517-83822003000100006

[69] Breznak JA , Costilow RN . 2014. Physicochemical factors in growth, p 309–329. In Methods for general and molecular microbiology. ASM Press, Washington, DC, USA. doi:10.1128/9781555817497

[70] Seo SW , Kim D , Szubin R , Palsson BO . 2015. Genome-wide reconstruction of OxyR and SoxRS transcriptional regulatory networks under oxidative stress in Escherichia coli K-12 Mg1655. Cell Rep 12:1289–1299. doi:10.1016/j.celrep.2015.07.043 26279566

[71] Durand S , Storz G . 2010. Reprogramming of anaerobic metabolism by the FnrS small RNA. Mol Microbiol 75:1215–1231. doi:10.1111/j.1365-2958.2010.07044.x 20070527 PMC2941437

[72] Boysen A , Møller-Jensen J , Kallipolitis B , Valentin-Hansen P , Overgaard M . 2010. Translational regulation of gene expression by an anaerobically induced small non-coding RNA in Escherichia coli. J Biol Chem 285:10690–10702. doi:10.1074/jbc.M109.089755 20075074 PMC2856277

[73] Ito K , Nakanishi M , Lee W-C , Sasaki H , Zenno S , Saigo K , Kitade Y , Tanokura M . 2005. Crystallization and preliminary X-ray analysis of AzoR (azoreductase) from Escherichia coli. Acta Crystallogr Sect F Struct Biol Cryst Commun 61:399–402. doi:10.1107/S1744309105007918 PMC1952434 16511052

[74] Punj S , John GH . 2009. Purification and identification of an FMN-dependent NAD(P)H azoreductase from Enterococcus faecalis. Curr Issues Mol Biol 11:59–65. doi:10.21775/cimb.011.059 18791262

[75] Morrison JM , Wright CM , John GH . 2012. Identification, isolation and characterization of a novel azoreductase from Clostridium perfringens. Anaerobe 18:229–234. doi:10.1016/j.anaerobe.2011.12.006 22182443

[76] Bardi L , Marzona M . 2010. Factors affecting the complete mineralization of azo dyes, p 195–210. In The handbook of environmental chemistry. doi:10.1007/698_2009_50

[77] Mani A , Hameed SAS . 2016. Accelerated production of oxygen-insensitive azoreductase from mutant Pseudomonas species for degradation azo dyes under aerobic condition. Asian J Chem 28:2562–2570. doi:10.14233/ajchem.2016.20113

[78] Clark DP . 1989. The fermentation pathways of Escherichia coli. FEMS Microbiol Rev 5:223–234. doi:10.1016/0168-6445(89)90033-8 2698228

[79] Jones SA , Chowdhury FZ , Fabich AJ , Anderson A , Schreiner DM , House AL , Autieri SM , Leatham MP , Lins JJ , Jorgensen M , Cohen PS , Conway T . 2007. Respiration of Escherichia coli in the mouse intestine. Infect Immun 75:4891–4899. doi:10.1128/IAI.00484-07 17698572 PMC2044527

[80] Jones SA , Gibson T , Maltby RC , Chowdhury FZ , Stewart V , Cohen PS , Conway T . 2011. Anaerobic respiration of Escherichia coli in the mouse intestine. Infect Immun 79:4218–4226. doi:10.1128/IAI.05395-11 21825069 PMC3187261

[81] Zhou Y , Imlay JA . 2020. Escherichia coli K-12 lacks a high-affinity assimilatory cysteine importer. mBio 11:e01073-20. doi:10.1128/mBio.01073-20 32518189 PMC7373191

[82] Braccia DJ , Jiang X , Pop M , Hall AB . 2021. The capacity to produce hydrogen sulfide (H_2_S) via cysteine degradation is ubiquitous in the human gut microbiome. Front Microbiol 12:705583. doi:10.3389/fmicb.2021.705583 34745023 PMC8564485

[83] Loubinoux J , Bronowicki J-P , Pereira IAC , Mougenel J-L , Faou AE . 2002. Sulfate-reducing bacteria in human feces and their association with inflammatory bowel diseases. FEMS Microbiol Ecol 40:107–112. doi:10.1111/j.1574-6941.2002.tb00942.x 19709217

[84] Rey FE , Gonzalez MD , Cheng J , Wu M , Ahern PP , Gordon JI . 2013. Metabolic niche of a prominent sulfate-reducing human gut bacterium. Proc Natl Acad Sci U S A 110:13582–13587. doi:10.1073/pnas.1312524110 23898195 PMC3746858

[85] Pitcher MC , Beatty ER , Cummings JH . 2000. The contribution of sulphate reducing bacteria and 5-aminosalicylic acid to faecal sulphide in patients with ulcerative colitis. Gut 46:64–72. doi:10.1136/gut.46.1.64 10601057 PMC1727787

[86] Barton LL , Ritz NL , Fauque GD , Lin HC . 2017. Sulfur cycling and the intestinal microbiome. Dig Dis Sci 62:2241–2257. doi:10.1007/s10620-017-4689-5 28766244

[87] Arnold LE , Lofthouse N , Hurt E . 2012. Artificial food colors and attention-deficit/hyperactivity symptoms: conclusions to dye for. Neurotherapeutics 9:599–609. doi:10.1007/s13311-012-0133-x 22864801 PMC3441937

[88] Jensen SI , Lennen RM , Herrgård MJ , Nielsen AT . 2015. Seven gene deletions in seven days: fast generation of Escherichia coli strains tolerant to acetate and osmotic stress. Sci Rep 5:17874. doi:10.1038/srep17874 26643270 PMC4672327

[89] Langmead B , Salzberg SL . 2012. Fast gapped-read alignment with Bowtie 2. Nat Methods 9:357–359. doi:10.1038/nmeth.1923 22388286 PMC3322381

[90] Anders S , Pyl PT , Huber W . 2015. HTSeq--a python framework to work with high-throughput sequencing data. Bioinformatics 31:166–169. doi:10.1093/bioinformatics/btu638 25260700 PMC4287950

[91] Ritchie ME , Phipson B , Wu D , Hu Y , Law CW , Shi W , Smyth GK . 2015. Limma powers differential expression analyses for RNA-sequencing and microarray studies. Nucleic Acids Res 43:e47. doi:10.1093/nar/gkv007 25605792 PMC4402510

[92] Kelly RT , Page JS , Luo Q , Moore RJ , Orton DJ , Tang K , Smith RD . 2006. Chemically etched open tubular and monolithic emitters for nanoelectrospray ionization mass spectrometry. Anal Chem 78:7796–7801. doi:10.1021/ac061133r 17105173 PMC1769309

[93] Zhang X , Smits AH , van Tilburg GB , Ovaa H , Huber W , Vermeulen M . 2018. Proteome-wide identification of ubiquitin interactions using UbIA-MS. Nat Protoc 13:530–550. doi:10.1038/nprot.2017.147 29446774

[94] Cline JD . 1969. Spectrophotometric determination of hydrogen sulfide in natural waters. Limnol Oceanogr 14:454–458. doi:10.4319/lo.1969.14.3.0454

[95] Thomason LC , Costantino N , Court DL . 2007. *E. coli* genome manipulation by P1 transduction, p 1. In Current protocols in molecular biology. John Wiley & Sons, Inc, Hoboken, NJ, USA. doi:10.1002/0471142727.mb0117s79 18265391

